# Metabolite Changes After Metabolic Surgery – Associations to Parameters Reflecting Glucose Homeostasis and Lipid Levels

**DOI:** 10.3389/fendo.2021.786952

**Published:** 2021-12-16

**Authors:** Sofie Ahlin, Consuelo Cefalo, Isabel Bondia-Pons, Kajetan Trošt, Esmeralda Capristo, Luca Marini, Montserrat Romero, Antonio Zorzano, Amalia Gastaldelli, Geltrude Mingrone, John J. Nolan

**Affiliations:** ^1^ Department of Molecular and Clinical Medicine, Institute of Medicine, The Sahlgrenska Academy at the University of Gothenburg, Gothenburg, Sweden; ^2^ Department of Medical and Surgery Sciences, Fondazione Policlinico Universitario A. Gemelli Istituto di Ricovero e Cura a Carattere Scientifico (IRCCS), Rome, Italy; ^3^ Research Department, Steno Diabetes Center, Gentofte, Denmark; ^4^ Departament de Bioquímica i Biomedicina Molecular, Facultat de Biologia, Universitat de Barcelona, Barcelona, Spain; ^5^ Institute for Research in Biomedicine (IRB Barcelona), Barcelona Institute of Science and Technology (BIST), Barcelona, Spain; ^6^ CIBERDEM, Centro de Investigación Biomédica en Red (CIBER) de Diabetes y Enfermedades Metabólicas Asociadas, Barcelona, Instituto de Salud Carlos III, Barcelona, Spain; ^7^ Cardiometabolic Risk Laboratory, Institute of Clinical Physiology, Consiglio Nazionale delle Ricerche (CNR), Pisa, Italy; ^8^ Department of Diabetes & Nutritional Sciences, Faculty of Life Sciences & Medicine, King’s College, London, United Kingdom; ^9^ Department of Clinical Medicine, Trinity College, Dublin, Ireland

**Keywords:** metabolic surgery, metabolomics, branched chain amino acids (BCAA), adipose tissue, metabolic pathway

## Abstract

**Aims:**

To test the hypothesis that adipose tissue gene expression patterns would be affected by metabolic surgery and we aimed to identify genes and metabolic pathways as well as metabolites correlating with metabolic changes following metabolic surgery.

**Materials and Methods:**

This observational study was conducted at the Obesity Unit at the Catholic University Hospital of the Sacred Heart in Rome, Italy. Fifteen patients, of which six patients underwent Roux-en-Y gastric bypass and nine patients underwent biliopancreatic diversion, were included. The participants underwent an oral glucose tolerance test and a hyperinsulinemic euglycemic clamp. Small polar metabolites were analyzed with a two-dimensional gas chromatography coupled to time-of-flight mass spectrometry (GC×GC-TOFMS). Gene expression analysis of genes related to metabolism of amino acids and fatty acids were analyzed in subcutaneous adipose tissue. All procedures were performed at study start and at follow-up (after 185.3 ± 72.9 days).

**Results:**

Twelve metabolites were significantly changed after metabolic surgery. Six metabolites were identified as 3-indoleacetic acid, 2-hydroxybutyric acid, valine, glutamic acid, 4-hydroxybenzeneacetic acid and alpha-tocopherol. The branched chain amino acids displayed a significant decrease together with a decrease in BCAT1 adipose tissue mRNA levels. Changes in the identified metabolites were associated to changes in lipid, insulin and glucose levels.

**Conclusions:**

Our study has identified metabolites and metabolic pathways that are altered by metabolic surgery and may be used as biomarkers for metabolic improvement.

**Graphical Abstract:**
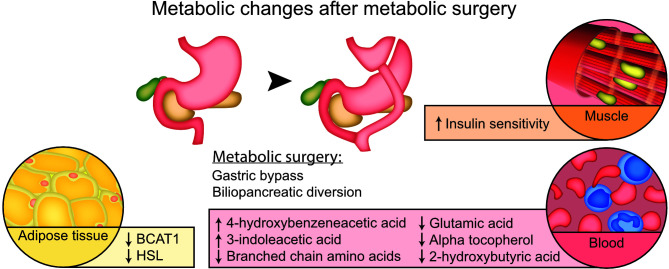


## Introduction

Treatment with metabolic surgery usually results in a long-term weigh loss (1) but is also associated with remission of type 2 diabetes, improvement of insulin resistance and prevention of cardiovascular disease (2–4). For some metabolic surgery procedures, where a part of the small intestine is bypassed, the improvement in glucose metabolism exceeds what is predicted and these benefits occur before the patients have started to lose weight (5, 6). This raises the possibility that there are additional mediators of the metabolic improvement after metabolic surgery other than weight loss.

Metabolites are the product of the activity of the genome, transcriptome and proteome and are thought to be the closest link to an organism’s phenotype (7, 8). Hence, metabolomics analysis is considered a very suitable method to investigate complex conditions such as type 2 diabetes and insulin resistance (9). However, how metabolites are regulated by different metabolic surgery procedures and how they are related to the metabolic improvement is less well understood.

Using an integrative approach to analyze adipose tissue gene expression in parallel with metabolite profiling, we aimed to identify metabolic pathways and metabolites related to the metabolic improvement after metabolic surgery.

## Materials and Methods

### Ethics Statement

The ethics committee at the Catholic University Hospital of the Sacred Heart, Rome, Italy, approved all study protocols. Participants gave informed consent to participate.

### Study Participants

Recruitment of participants was performed at the Catholic University Hospital of the Sacred Heart in Rome, Italy. In total 15 patients were recruited of which six patients underwent Roux-en-Y gastric bypass (RYGBP) and nine patients underwent biliopancreatic diversion (BPD) as treatment for obesity. The surgeon decided the surgical procedure for each participant. Usually BPD is used for patients with higher BMI (>45 kg/m^2^) (10). Inclusion criteria were a body mass index of 40 kg/m^2^ or more, no previous diagnosis of type 2 diabetes with a HbA1c ≤ 7% and willingness to participate in the study. Major endocrine disease, malignancies and liver cirrhosis were established as exclusion criteria. Follow-up visits were performed after 185.3 ± 72.9 days.

Blood samples from participants in a fasting state were collected together with anthropometry both at study start and at follow-up. All participants also underwent an oral glucose tolerance test. Blood chemistry was performed at the central laboratory of the Catholic University Hospital of the Sacred Heart in Rome, Italy.

### Hyperinsulinemic-Euglycemic Clamp

Peripheral insulin sensitivity was assessed by a hyperinsulinemic-euglycemic clamp in participants that had fasted overnight (11), which has previously been described in detail (12). At the start of the test, two venous accesses were established, one in the antecubital vein for infusions and another was inserted into a hand vein. The hand was heated in a heated air box set at 60°C to obtain arterialized blood samples. A primed constant insulin infusion at the rate of 6 pmol/min/kg was given to the participant together with a variable glucose infusion that was adjusted every 5 minutes on the basis on blood glucose measurements so that blood glucose concentration was stable at the fasting value for 2 h. The last 40 minutes of the test was used to calculate whole body glucose uptake (M, mg/kg/min). All samples were stored at – 80°C before further analysis.

### Other Measurements of Insulin Sensitivity and Insulin Secretion

Homeostatic model assessment of insulin resistance (HOMA-IR) (13) and oral glucose insulin sensitivity (OGIS) (14) were calculated. Insulin secretion was estimated with C-peptide deconvolution (15, 16).

### Adipose Tissue Gene Expression

Subcutaneous adipose tissue biopsies were obtained from participants before at study start and at follow-up. The biopsies were taken from the umbilical area, snap frozen in liquid nitrogen and stored in -80°C until further analysis. Analysis of mRNA levels of genes related to the metabolism of branched chain amino acids, fatty acid metabolism and amino acid metabolism were performed. mRNA isolation was performed using magnetic beads. Subcutaneous white adipose tissue was sorted into lysis buffer. Directly after tissue sorting, the plate containing lysis buffer and lysed tissue was incubated for 15 minutes at 65°C. Subsequently, RNA was purified using RNA Clean XP bead suspension (Agencourt Bioscience). RNA was quantified using the Quant-iT RNA Assay kit (Life Technologies), and after that cDNA synthesis was performed by Pico Profiling. mRNA isolation and cDNA synthesis was carried out at Functional Genomics Platform of IRB Barcelona (17).

Quantitative real-time PCR was performed using the ABI Prism 7900 HT real-time PCR machine (Applied Biosystems) and the SYBR Green PCR Master Mix (Applied Biosystems), using Peptidylprolyl Isomerase A (PPIA) as internal controls for normalization. The following genes involved in branched chain amino acid metabolism were analyzed: branched chain amino acid transaminase 1 (BCAT1), branched chain amino acid transaminase 2 (BCAT2), Branched chain ketoacid dehydrogenase α (BCKDHα) and branched chain ketoacid dehydrogenase β (BCKDHβ). Additional gene expression analysis of genes related to amino acid metabolism were also performed for the following genes: glutamate dehydrogenase (GDH), asparagine synthetase (AS), glycine amidinotransferase (AT); and for the following genes related to fatty acid metabolism: acetyl-CoA carboxylase α (ACCα), patatin like phospholipase domain containing 2 (ATGL), Fatty acid synthase (FASN), Hormone sensitive lipase (HSL), diacylglycerol O-acyltransferase 1 (DGAT1), perilipin 1 (PLIN1), perilipin 2 (PLIN2). For primer sequences, please, see **Supplemental Table S1**.

### Small Polar Metabolites by Two-Dimensional Gas Chromatography Coupled to Time-of-Flight Mass Spectrometry (GC×GC-TOFMS)

Small polar metabolites were analyzed with two-dimensional gas chromatography coupled to time-of-flight mass spectrometry (GC×GC-TOFMS). Proteins from 30 μl plasma were precipitated using 400 μl methanol. Then 10 μl of group specific internal standards (heptadecanoic acid-d33 (175.36 mg/l), valine-d8 (35.72 mg/l), succinic acid-d4 (58.54 mg/l) and glutamic acid-d5 (110.43 mg/l)) were added and the samples were vortexed and left to precipitate on ice for 30 min. Half of the supernatant was evaporated to dryness after centrifugation for 3 min at 10000 rpm. An MPS autosampler (Gerstel, Mülheim an der Ruhr, Germany) was used to derivatize evaporated samples. The two step- derivatization started by adding 25 μl of methoxyamine hydrochloride (98%) and the samples were incubated for 1 hour at 45°C. Then, 25 μl of N-methyl-N-trimethylsilyltrifluoroacetamide was added as a second step in the derivatization and the samples were again incubated for 1 hour at 45°C. Retention index standards (n-alkanes, 25 μl, 8 mg/l) and an injection standard (4,4′-dibromooctafluorobiphenyl in hexane, 50 μl, 9.8 mg/l) were added and 1 μl of the derivatized sample was injected into the system (Pegasus 4D from Leco, St. Joseph, MI, USA).

A system of 3 columns were used for chromatographic separation. The first column was a 1.7 m deactivated retention gap column (0.53 mm ID, FS deactivated, Agilent technologies, USA) followed by a 10 m × 0.18 mm I.D. Rxi-5 ms (Restek Corp., Bellefonte, PA, USA) primary column and a 1.5 m × 0.1 mm I.D. BPX-50 (SGE Analytical Science, Austin, TX, USA) secondary column. The temperature program included the following steps: 50°C for 2 minutes followed by a gradually increasing temperature by steps of 7°C/minute up to 240°C and finally 25°C/minute to 300°C where it was held for 3 min. The secondary column had a slightly different temperature program that was maintained 20°C higher than in the primary column.

Raw data were processed with ChromaTOF version 4.32 (LECO Corporation, St. Joseph, MI). Signal to noise values higher than 100 was used as an inclusion criterion. The area of selected ions was used for target compounds and the total ion chromatogram area was used for untargeted compounds. External calibration curves with at least 6 concentration points were used to quantify selected compounds. Results were then exported as text files for further processing with Guineu software (18).

The samples were aligned and normalized with internal standards. Compounds belonging to chemical background or outside of the linear range were excluded. Small polar metabolites were identified using an in-house library, the NIST14 mass spectral library, and the Golm metabolome database (19). Metabolites verified with the in-house library were assigned as class 1, metabolites reaching similarity higher than 850 and a retention index difference lower than 30 were assigned to class 2. Other metabolites which did not meet the previous requirement were assigned as class 3 and were characterized by their spectra and Golm group annotation.

The coefficient of variation of compounds quantified by external calibration curve and measured on quality control plasma was on average 11% (n=8). The coefficient of variation of spiked compounds across the sample set also reached on average 11% (n=36). Metabolites that were present in more than 80% of the samples were included into further statistical analysis.

### Statistical Analysis

Baseline characteristics are presented as means ± standard deviations (SD) or numbers and percent. Paired t-tests were used to analyze differences at study start and at follow-up. Student’s t-tests were used to analyze differences between the two surgery groups at study start and at follow-up. Fisher’s exact test was used for comparisons between the surgery groups of categorical data. Data on anthropometry and metabolic parameters were log2-transformed to achieve normal distribution. Metabolomics data were normalized by using log2-transformed values and scaled into zero mean and unit variance (autoscaling) (20) to obtain metabolite profiles comparable to each other. P-value < 0.05 was considered statistically significant. For the metabolomics data a false discovery rate of q-value < 0.05 was for correction of multiple comparisons. Spearman correlation analysis was performed on fold changes of relative concentrations (after versus before metabolic surgery) of metabolomic data and metabolic parameters to select variables for linear regression analysis. The fold changes were then log2-transformed (log2(post/pre)) to achieve linear relationships before linear regression analysis. For statistical analysis of gene expression, gene expression was normalized to mean value at study start and differences at study start and at follow-up were calculated with paired T-test. All statistical analyses were performed by using the R Project for Statistical Computing version 3.x. and IBM SPSS statistics software 21.0 (IBM Corp. NY. USA).

## Results

### Participants’ Characteristics at Study Start and Follow-Up

Participants’ characteristics at study start and at follow-up are presented in **Table 1**. The participants treated with biliopancreatic diversion (BPD) were different from the participants treated with Roux-en Y gastric bypass (RYGB) in some aspects at study start. All patients treated with RYGB were men (n = 6, 100%) compared to four men of nine included patients in the BPD group (44.4%, p-value 0.044). The patients in the RYGB group also had significantly lower BMI compared to the BPD group (45.3 kg/m^2^ ± 5.7 vs 55.9 kg/m^2^ ± 9.7, p-value 0.034.

**Table 1:** Participants’ characteristics at study start and at follow-up visit.

	ALL		BPD		RYGB		% differences		
	At study start	At follow-up	At study start	At follow-up	At study start	At follow-up	ALL	BPD	RYGB
Number	15	15	9	9	6	6	15	9	6
Days until follow-up		185.3 ± 72.9		183.7 ± 61.8		187.8 ± 93.6			
Men, n (%)	10 (66.7)	–	4 (44.4) *	–	6 (100) *	–	–	–	–
Age, years	44.3 ± 8.3	–	44.7 ± 8.1	–	43.7 ± 9.4	–	–	–	–
Weight, kg	151.0 ± 24.8 ^###^	110.4 ± 22.2 ^###^	156.3 ± 28.7 ^###^	109.4 ± 26.8 ^###^	143.0 ± 16.7 ^##^	112.0 ± 15.1 ^##^	-26.5 ± 10.8	-29.8 ± 12.0	21.6 ± 7.0
% Excess weight loss	–	53.3 ± 19.9	–	55.5 ± 20.9	–	50.0 ± 19.7	–	–	–
BMI, kg/m^2^	51.7 ± 9.7 ^###^	37.7 ± 7.7 ^###^	55.9 ± 9.7 * ^###^	39.1 ± 8.9 ^###^	45.3 ± 5.7 * ^##^	35.5 ± 5.5 ^##^	-26.1 ± 11.4	-29.2 ± 13.0	-21.6 ± 7.0
Change in BMI	–	-14.0 ± 7.2	–	-16.9 ± 7.9 *	–	-9.7 ± 3.1 *	–	–	–
Glucose, mg/dl	97.5 ± 17.0 ^###^	76.8 ± 11.1 ^###^	101.7 ± 20.7 ^#^	76.7 ± 13.8 ^#^	91.3 ± 6.3 ^##^	77.0 ± 6.3 ^##^	-19.8 ± 13.9	-22.6 ± 17.1	-15.6 ± 6.3
Total cholesterol, mg/dl	189.6 ± 28.2 ^###^	138.1 ± 28.6 ^###^	197.0 ± 31.3 ^###^	124.7 ± 17.3 * ^###^	178.5 ± 20.2	158.2 ± 31.2 *	-25.0 ± 22.1	-35.2 ± 13.7 *	-9.8 ± 24.7 *
HDL-cholesterol, mg/dl	47.1 ± 9.5	41.7 ± 12.3	47.9 ± 7.9	38.9 ± 13.2	46.0 ± 12.4	45.5 ± 11.0	-8.0 ± 38.5	-14.0 ± 42.1	0.5 ± 35.5
LDL-cholesterol, mg/dl	119.5 ± 22.1 ^##^	74.9 ± 27.5 ^##^	127.9 ± 16.3 ^##^	61.6 ± 14.2 * ^##^	107.8 ± 25.6	92.7 ± 32.0 *	-37.8 ± 23.9	-50.0 ± 15.2 *	-20.8 ± 24.4 *
Triglycerides, mg/dl	134.9 ± 58.5	109.6 ± 49.3	141.4 ± 52.7	116.2 ± 61.1	126.3 ± 69.7	99.7 ± 25.2	-8.7 ± 46.2	-16.4 ± 19.6	1.7 ± 69.2
Type 2 diabetes	2	0	1	0	1	0	–	–	–
HbA1c, mmol/mol	41.4 ± 3.7 ^###^	36.5 ± 2.6 ^###^	42.1 ± 4.0 ^#^	37.3 ± 2.5 ^#^	40.3 ± 3.3 ^##^	35.3 ± 2.3 ^##^	-11.3 ± 8.1	-10.7 ± 9.8	-12.2 ± 5.3
Whole body glucose uptake, M, mg/kg/min	2.7 ± 1.4 ^###^	6.0 ± 2.2 ^###^	2.3 ± 1.2 ^###^	6.1 ± 2.3 ^###^	3.3 ± 1.7 ^#^	5.7 ± 2.3 ^#^	151.0 ± 116.5	188.2 ± 119.4	95.2 ± 94.6
HOMA-IR	3.3 ± 2.2 ^##^	1.1 ± 0.6 ^##^	3.0 ± 2.2	1.3 ± 0.6	3.6 ± 2.3 ^#^	0.9 ± 0.5 ^#^	-42.7 ± 62.4	-25.4 ± 74.7	-68.6 ± 25.5
Insulin secretion, pmol/min/m^2^	223.4 ± 100.7	196.6 ± 140.0	226.2 ± 89.9	173.9 ± 98.0	219.2 ± 124.2	230.6 ± 192.9	-14.3 ± 37.1	-20.4 ± 37.6	-5.1 ± 37.8

Data are presented as mean values ± standard deviation or numbers and percent. * = p-values < 0.05 in comparisons between the BPD group and the RYGB group at study start and follow-up.^#^ = p-values < 0.05, ^##^ = p-values < 0.01 and ^###^ = p-values < 0.001 in comparisons at study start and at follow-up in each study group. BMI, Body Mass Index; HDL-cholesterol, high density lipoprotein cholesterol; LDL-cholesterol, low density lipoprotein cholesterol; HbA1c, glycated hemoglobin; HOMA-IR, Homeostatic Model Assessment for Insulin Resistance.

Indices for adiposity (weight, BMI) and parameters reflecting glucose homeostasis (fasting glucose, HbA1c and whole-body glucose uptake) were significantly improved after metabolic surgery for both the RYGB and BPD group (**Table 1**). Total serum cholesterol and S-LDL-cholesterol were significantly reduced after intervention in the BPD group, but this was not observed in the RYGB group (**Table 1**).

### Small Polar Metabolites

In total, 378 small polar metabolites were observed in the GC×GC-TOF-MS database after normalization and filtering. Out of them, 73 metabolites were identified, mainly as amino acids, organic acids and fatty acids.

Twelve metabolites were significantly (p-value < 0.05; q-value < 0.05) altered at follow-up compared with the study start (pre vs post) in the whole study population (**Table 2**). Six of these 12 metabolites could be identified as 3-indoleacetic acid, 2-hydroxybutyric acid, valine, glutamic acid, 4-hydroxybenzeneacetic acid and alpha-tocopherol. 3-indoleacetic acid and 4-hydroxybenzeneacetic acid displayed an increase at follow-up whereas the other identified metabolites were decreased. None of the small polar metabolites displayed a significant difference between the BPD and RYGBP group at study start or at follow-up.

**Table 2:** Small polar metabolites with signficant change between study start and follow-up in the total surgery group (n = 15).

Metabolite (relative concentration)	At study start	At follow-up	p-value	q-value
ID no. 400	6.45 ± 1.56	39.89 ± 37.04	0.00012	0.026
3-indoleacetic acid	57.34 ± 53.48	309.37 ± 362.84	0.00016	0.018
ID no. 493	9.31 ± 4.88	17.71 ± 5.79	0.00018	0.013
ID no. 361	7.21 ± 3.63	37.23 ± 35.10	0.00022	0.012
ID no. 294	565.07 ± 194.45	349.27 ± 194.06	0.00030	0.013
2-hydroxybutyric acid	399.49 ± 209.60	164.03 ± 85.83	0.00039	0.014
Valine	574.80 ± 131.67	395.01 ± 109.86	0.00075	0.023
ID no. 296	182.64 ± 66.16	343.78 ± 144.47	0.0012	0.033
ID no. 580	13.90 ± 9.77	32.91 ± 17.23	0.0016	0.039
Glutamic acid	244.25 ± 85.84	141.77 ± 80.66	0.0017	0.037
4 hydroxybenzeneacetic acid	22.83 ± 31.43	152.01 ± 164.83	0.0017	0.034
Alpha tocopherol	28.75 ± 9.11	19.62 ± 8.40	0.0019	0.036

The data are presented as mean relative concentration ± standard deviation. Paired T-test on log2-transformed values was used to analyse differences in metabolite levels at study start and follow-up. False discovery rate was used to adjust for multiple testing.

### Changes in GSG Index, Branched Chain Amino Acids, Aromatic Amino Acids and Saturated Fatty Acids After Metabolic Surgery

We also analyzed changes in groups of metabolites known to be associated to metabolic diseases (21) such as branched chain amino acids (BCAA = leucine, valine, isoleucine), aromatic amino acids (AAA = phenylalanine + tyrosine + tryptophan), GSG index [calculated from amino acids involved in oxidative stress and the synthesis of glutathione, Glutamate/(Serine+Glycine)], a marker of liver fibrosis in non-alcoholic fatty liver disease (NAFLD) (22) and saturated fatty acids (Palmitic acid + Stearic acid) at study start and at follow-up.

GSG index and BCAA displayed a significant decrease after metabolic surgery (p-value 0.0004 and p-value 0.004, respectively, **Table 3**). No differences in GSG-index, BCAA, AAA or saturated fatty acids were observed between the BPD group and RYGB group at study start but at follow-up the RYGB group displayed a significantly lower levels of AAA (p-value 0.006) compared with the BPD group (p-value 0.006, **Table 3**).

**Table 3:** Changes in GSG index, branched chain amino acids, aromatic amino acids and saturated fatty acids after metabolic surgery.

	ALL		BPD		RYGB	
	At study start	At follow-up	At study start	At follow-up	At study start	At follow-up
GSG-index	0.69 ± 0.26 ^###^	0.31 ± 0.20 ^###^	0.66 ± 0.25 ^#^	0.34 ± 0.23 ^#^	0.73 ± 0.29 ^#^	0.28 ± 0.14 ^#^
Branched chain amino acids	1243.65 ± 341.90 ^##^	888.37 ± 283.09 ^##^	1129.64 ± 199.39	981.62 ± 285.96	1414.67 ± 453.01 ^###^	748.50 ± 233.30 ^###^
Aromatic amino acids	415.81 ± 98.78	345.50 ± 117.68	397.24 ± 107.24	405.40 ± 80.22 **	443.67 ± 85.85 ^#^	255.65 ± 111.07 ** ^#^
Saturated fatty acids	5007.40 ± 866.23	4501.13 ± 1374.79	5282.23 ± 752.38	4882.31 ± 1359.03	4595.15 ± 924.87	3929.37 ± 1297.38

** = p-values < 0.01 in comparisons between the BPD group and the RYGB group at study start and follow-up. ^#^ = p-values < 0.05, ^##^ = p-values < 0.01 and ^###^ = p-values < 0.001 in comparisons study start vs follow-up in each study group.

### Associations Between the Changes of Small Polar Metabolites and Changes in Metabolic Parameters

**Figure 1:**
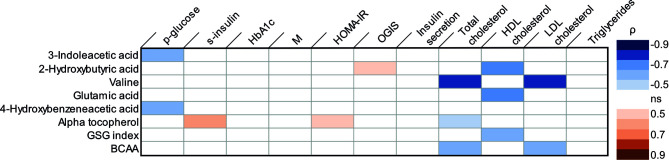
Correlations between changes in metabolic parameters [log2 (post/pre)] and changes in metabolites [log2 (post/pre)] in Spearman correlation analyses. Blue color indicates a significant negative correlation coefficient and red color a significant positive correlation coefficient. HbA1c, glycated hemoglobin; M, whole body glucose uptake; OGIS, oral glucose insulin sensitivity; HDL-cholesterol, High density lipoprotein cholesterol; LDL, low density lipoprotein cholesterol; BCAA, branched chain amino acids. ns, not significant.

Several correlations between parameters reflecting glucose homeostasis and the metabolites regulated by metabolic surgery were observed in Spearman correlation analyses (**Figure 1**). The change in 3-indoleacetic acid and the change in 4-hydroxybenzeneacetic acid were negatively correlated to the change in glucose. The changes in alpha-tocopherol was positively correlated to both changes in s-insulin and changes in HOMA-IR. 2-hydroxyburyric acid was correlated to OGIS. In linear regression analysis the change in 3-indoleacetic acid and the change in 4-hydroxybenzeneacetic acid could predict the change in glucose (R^2^ = 0.745; p-value<0.001 and R^2^ = 0.709; p-value 0.003; **Supplemental Figures S1A, B**). In addition, alpha tocopherol could significantly predict the change in s-insulin (R^2^ = 0.263; p-value 0.050; **Supplemental Figure S1C**) but could not predict the change in HOMA-IR (R^2^ = 0.253; p-value 0.056) and 2-hydroxybutyric acid could not predict the change in OGIS in a linear regression analysis (R^2^ = 0.235; p-value 0.067). No changes in metabolites were correlated to changes in insulin sensitivity estimated with whole body glucose uptake.

Correlations were also observed between changes in serum lipid levels and metabolites regulated by metabolic surgery (**Figure 1**). The change in valine, alpha-tocopherol and branched chain amino acids were all negatively correlated to changes in s-cholesterol. Changes in 2-hydroxybutyric acid, glutamic acid and GSG-index were negatively correlated to changes in HDL-cholesterol. In addition, the change in valine and branched chain amino acids also displayed negative correlations to the change in LDL-cholesterol. A linear regression model further established that the change in valine (R^2^ 0.0609; p-value 0.001), alpha-tocopherol (R^2^ 0.374; p-value 0.015) and branched chain amino acid (R^2^ 0.594; p-value 0.001) could predict the change in s-cholesterol (**Supplemental Figure S2** panel A–C) and that changes in 2-hydroxybutyric acid (R^2^ 0.672; p-value 0.001), glutamic acid (R^2^ 0.668; p-value 0.001) and GSG-index (R^2^ 0.534; p-value 0.007) could predict the change in HDL-cholesterol (**Supplemental Figure S2**, panel D–F). Furthermore, changes in valine (R^2^ 0.712; p-value 0.001) and branched chain amino acids (R^2^ 0.688; p-value 0.001) could predict the change in LDL-cholesterol (**Supplemental Figure S2** panel G, H).

### Adipose Tissue Gene Expression Analysis

**Figure 2:**
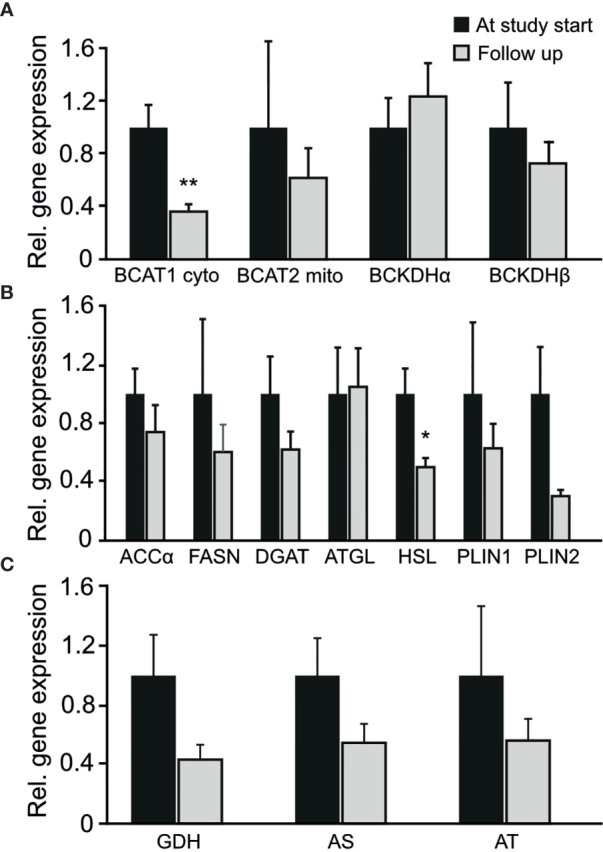
Changes in the entire cohort in relative gene expression of adipose tissue genes involved in **(A)** branched chain amino acid metabolism **(B)** fatty acid metabolism **(C)** amino acid metabolism. *p-value < 0.05. **p-value < 0.01.

Among the genes involved in branched chain amino acid metabolism BCAT1 displayed a significant reduction in mRNA levels in adipose tissue after surgery (p-value 0.003, **Figure 2A**). The changes in BCAT1 gene expression (log2(post/pre)) was not correlated to the changes in BCAA concentrations (log2(post/pre) (correlation coefficient -0.282; p-value 0.309). The gene expression of HSL (Hormone sensitive lipase), was also reduced in adipose tissue after metabolic surgery (p-value 0.017, **Figure 2B**). No significant changes in gene expression of genes related to amino acid metabolism (GDH, AS and AT) were observed (**Figure 2C**).

## Discussion

In this study we have investigated metabolic pathways and metabolites that are altered after metabolic surgery and related to the metabolic improvement by using an integrative approach analyzing adipose tissue gene expression in parallel with plasma metabolite profiling.

In our study branched chain amino acids, as well as glutamic acid, were down regulated after metabolic surgery, in line with previous studies (23–25). Serum levels of both BCAA as well as glutamic acid have previously been associated with insulin resistance (26–28). However, the changes in valine and BCAA or glutamic acid were not able to predict the changes in parameters reflecting glucose homeostasis in our study. Instead, changes in valine, BCAA and glutamic acid could predict changes in serum cholesterol levels. Interestingly, BCAAs have been shown to influence hepatic lipid metabolism both *in vitro* and *in vivo* (29), which may explain our observed association.

The previous reported links between circulating BCAA and metabolic function (26–28) have highlighted the importance of well-functioning BCAA metabolism. It has been suggested that elevated levels of branched chain amino acid associated with obesity could be a consequence of alterations of the BCAA catabolizing enzymes in adipose tissue (30). A recent report showed that BCAA catabolizing enzymes in visceral adipose tissue are decreased in women with obesity (31), which might account for the increased circulating levels of BCAA observed in the obese state. However, our results do not support this hypothesis. Instead we observed a decrease in adipose tissue BCAT1 gene expression in adipose tissue after metabolic surgery, which are in line with a study reporting that a decrease of mRNA *BCAT1* in the adipose tissue is a marker for weight loss maintenance after a low calorie diet (32).

Branched chain amino acids can downregulate the synthesis of the 3-indoleacetic acid in bacteria (33) present in in the gut (34). Thus, it is possible that the decreased levels of branched chain amino acids after metabolic surgery could result in the increased levels of 3-indoleacetic observed in our study. 3-indoleacetic acid is produced by tryptophan and a recent study has suggested that changes in indole tryptophan metabolites from gut bacteria can be responsible for the beneficial metabolic effects of metabolic surgery (35). This is an interesting hypothesis as the change in 3-indoleacetic acid could predict the change in circulating glucose levels in our study.

Additional three individual metabolites were regulated by metabolic surgery in our study: 2-hydroxybutyric acid, a derivate from alpha-ketobutyrate that is produced in the liver by the catabolism of threonine and methionine and glutathione synthesis, and alpha-tocopherol that both were decreased after metabolic surgery and 4-hydroxybeneacetic acid, produced from tyrosine in bacteria in the gut (34), which was increased. The decrease of 2-hydroxybutyric acid after metabolic surgery was correlated to HOMA-IR in a previous study (36). However, in our study the change of 2-hydroxybutyric acid was not associated with any of the parameters reflecting glucose homeostasis. A more interesting finding was the association of 4-hydroxybenzeneacetic acid to changes in glucose levels and the association between alpha tocopherol and fasting insulin levels. Furthermore, regulation of 4-hydroxybenzeneacetic acid after metabolic surgery has, to the best of our knowledge, not been reported before. Hence, both alpha tocopherol and 4-hydroxybezeneacetic acid may be interesting targets for future studies on glucose homeostasis after metabolic surgery.

A decrease in hormone sensitive lipase (HSL) was also observed after metabolic surgery in our study. A decrease of adipose tissue HSL mRNA levels after weight loss has been reported in women with obesity (37) whereas others report a reduction of HSL adipose tissue mRNA levels in obesity (38). The mechanism behind the reduction in HSL mRNA levels in adipose tissue in our study is unclear, but the promoter region of the HSL gene contains a glucose responsive region (39) and the improved insulin sensitivity and reduction in glucose levels after metabolic surgery may have an impact on HSL gene expression levels.

Our study has limitations. The study contains a relatively small number of patients, and it is reasonable to believe that there are other metabolite changes after metabolic surgery that we are not able to demonstrate here due to limited statistical power. In addition, no major differences in metabolites between the two surgery types were observed. This is probably due to a small number of patients in each group so the study did not have power enough to detect such differences. However, the two surgical procedures studied here, RYGB and BPD are different in some aspects such as differences in fat malabsorption and the weight loss achieved (40). Hence, it is reasonable to believe that some differences in metabolites might be detected but our study did not have power to detect such differences.

We here show that plasma concentrations of branched chain amino acids and adipose tissue mRNA levels of BCAT1 were down-regulated after metabolic surgery. In addition, results from this study have identified some new candidates, such as 4-hydroxybenzeneacetic acid, 2-hydroxybutyric acid and alpha tocopherol that may be used as biomarkers for the early metabolic improvement after metabolic surgery, which include short-term metabolic changes related to caloric restriction and improved hepatic insulin resistance as well as enteroendocrine changes (41). Future studies are needed to further validate our results in other study cohorts and with longer follow-up.

## Data Availability Statement

The datasets presented in this article are not readily available because for reasons of privacy. Anonymous data are available from the corresponding author on reasonable request. Requests to access the datasets should be directed to Sofie Ahlin, sofie.ahlin@gu.se.

## Ethics Statement

The studies involving human participants were reviewed and approved by the ethics committee at the Catholic University Hospital of the Sacred Heart, Rome, Italy. The patients/participants provided their written informed consent to participate in this study.

## Author Contributions

CC, EC, and LM collected data. AZ and MR. coordinated and performed the gene expression analysis. IB-P and KT coordinated and performed the metabolomics lab analysis. SA, AG, GM, JN, and AZ data interpretation. SA and GM wrote the manuscript. GM, JN, and AZ are responsible for study design. All authors contributed to discussion and reviewed/edited the manuscript. All authors contributed to the article and approved the submitted version.

## Funding

This study was funded by The Catholic university hospital of the Sacred heart, the Health & Medical Care Committee of the Region Västra Götaland (grant no. VGFOUREG-931560), the Swedish research council (grant no. 2016-00522), and the Magnus Bergvall Foundation (grant no 2019-03489). IB-P is grateful to the Novo Nordisk Foundation for her Clinical Research Fellow grant.

## Conflict of Interest

EC reports payment for educational events in collaborations with Novo Nordisk and Bruno Pharmaceutics.

The remaining authors declare that the research was conducted in the absence of any commercial or financial relationships that could be construed as a potential conflict of interest.

## Publisher’s Note

All claims expressed in this article are solely those of the authors and do not necessarily represent those of their affiliated organizations, or those of the publisher, the editors and the reviewers. Any product that may be evaluated in this article, or claim that may be made by its manufacturer, is not guaranteed or endorsed by the publisher.
