# Supplementary material for: Metabolite Changes After Metabolic Surgery – Associations to Parameters Reflecting Glucose Homeostasis and Lipid Levels
Source: Front Endocrinol (Lausanne). 2021 Dec 16;12:786952. doi: 10.3389/fendo.2021.786952 (PMC8716486; doi:10.3389/fendo.2021.786952)

Supplementary Material

| **Table of contents** | Page |
| --- | --- |
| **Supplemental table S1**. The SYBR Green primer sequences used for adipose tissue gene expression analysis. | 2 |
| **Supplemental figure S1**. Associations between changes in small polar metabolites and changes in parameters reflecting glucose homeostasis | 3 |
| **Supplemental figure S2**. Associations between changes in small polar metabolites and changes in serum lipid levels | 4-5 |

**Supplemental table S1. The SYBR Green primer sequences used for adipose tissue gene expression analysis.**

| Gene | Forward primer | Rev primer |
| --- | --- | --- |
| BCAT1 | GTGGAGTGGTCCTCAGAGTTT | AGCCAGGGTGCAATGACAG |
| BCAT2 | GCTCAACATGGACCGGATG | CCGCACATAGAGGCTGGTG |
| BCKDHα | CTACAAGAGCATGACACTGCTT | CCCTCCTCACCATAGTTGGTC |
| BCKDHβ | GTGCCTTGGATAACTCATTGGC | AGTGCATCTAAAGACTCCACCA |
| ACCα | ATGTCTGGCTTGCACCTAGTA | CCCCAAAGCGAGTAACAAATTCT |
| FASN | AAGGACCTGTCTAGGTTTGATGC | TGGCTTCATAGGTGACTTCCA |
| DGAT | TATTGCGGCCAATGTCTTTGC | CACTGGAGTGATAGACTCAACCA |
| ATGL | GGCTTCCTCGGCGTCTACTA | TTTACCAGGTTGAAGGAGGGG |
| HSL | TCAGTGTCTAGGTCAGACTGG | AGGCTTCTGTTGGGTATTGGA |
| PLIN1 | TGTGCAATGCCTATGAGAAGG | AGGGCGGGGATCTTTTCCT |
| PLIN2 | ATGGCATCCGTTGCAGTTGAT | GGACATGAGGTCATACGTGGAG |
| GDH | TCGTGGAGGACAAGTTGGTG | TTGCAGGGCTTGATGATCCG |
| AS | AAGAGGGACACCAATAGCAAAAA | GCAGAACGTAAGGCTTTCCAT |
| AT | GTGATGGCACTATGCACCTAC | TTCACGGATGCAGTTGACACC |

**Supplemental figure S1**. Associations between changes in small polar metabolites and changes in parameters reflecting glucose homeostasis in linear regression analysis. **A.** log2 FC 3-indoleacetic acid vs log2 glucose. **B**. log2 FC 4-hydroxybenzeneacetic acid vs log2 FC glucose. **C**. log2 FC alpha-tocopherol vs log2 FC fasting insulin.


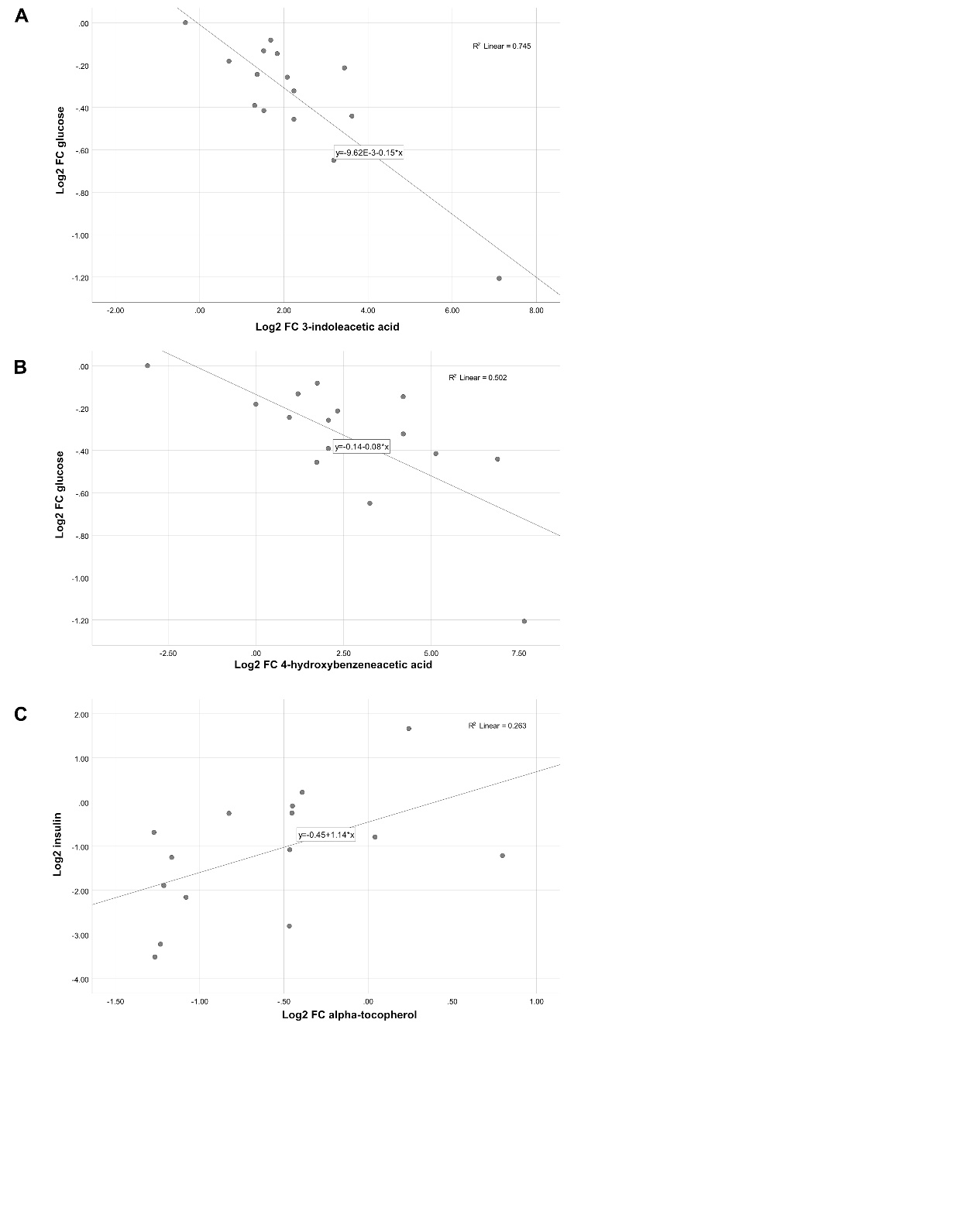


**Supplemental figure S2**. Associations between changes in small polar metabolites and changes in serum lipid levels in linear regression model. **A**. log2 FC valine versus log2 FC total s-cholesterol. **B**. log2 FC alpha-tocopherol versus log2 FC total s-cholesterol **C**. log2 FC BCAA versus log2 FC total s-cholesterol. **D**. log2 FC 2-hydroxybutyric acid versus log2 FC s-HDL-cholesterol. **E**. log2 FC glutamic acid versus log2 FC s-HDL-cholesterol. **F.** log2 FC GSG index versus log2 FC s-HDL-cholesterol. **G**. log2 FC valine versus log2 FC s-LDL-cholesterol. **H.** log2 FC BCAA versus log2 FC s-LDL-cholesterol FC = fold changes. BCAA = branched chain amino acids.


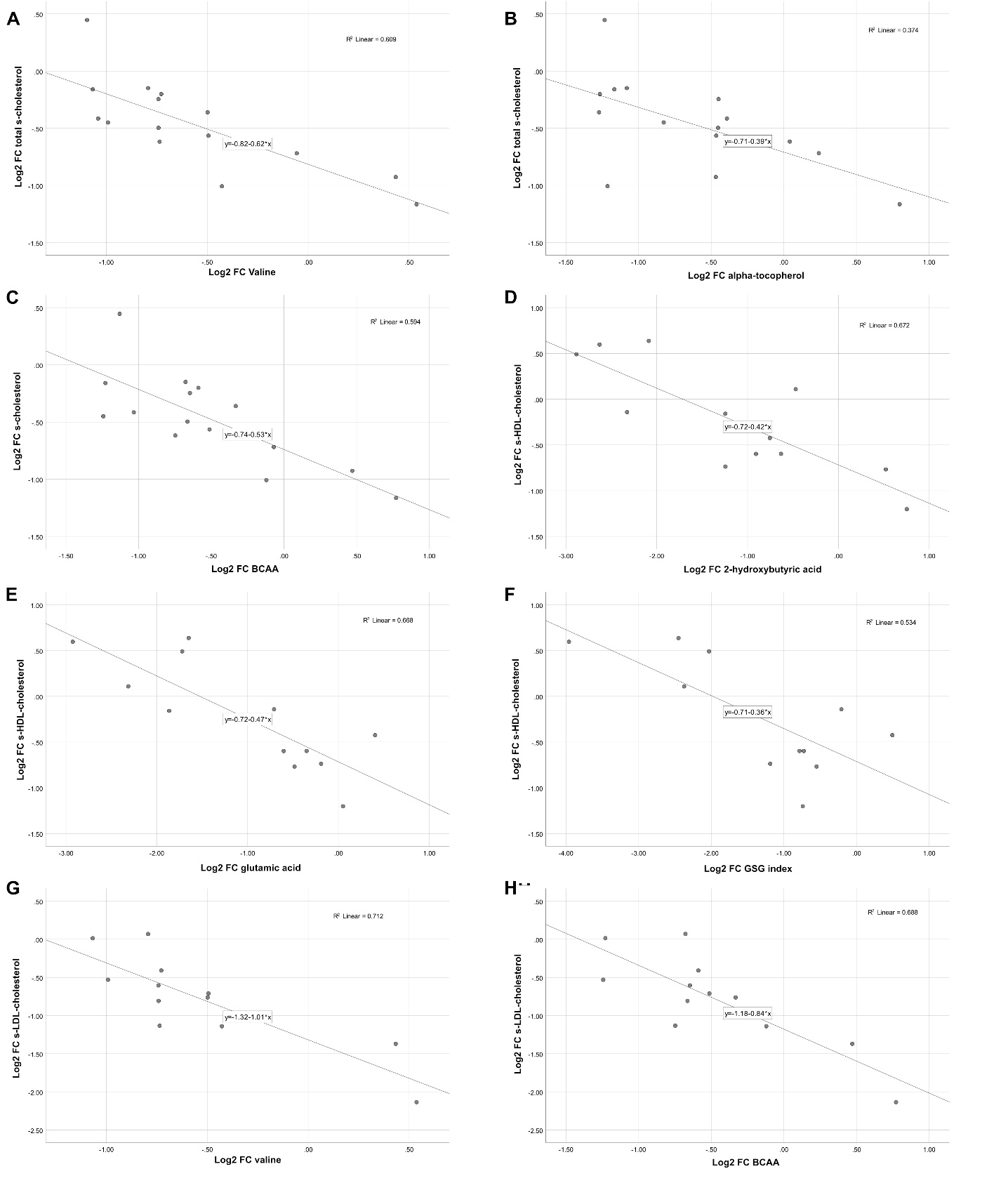

Supplement: Supplementary file 1 [file DataSheet_1.docx]
